# Cost analysis of skeletal-related events in Spanish patients with bone metastases from solid tumours

**DOI:** 10.1007/s12094-013-1077-2

**Published:** 2013-08-13

**Authors:** I. Durán, C. Garzón, A. Sánchez, I. García-Carbonero, J. L. Pérez-Gracia, M. Á. Seguí-Palmer, R. Wei, G. Restovic, J. A. Gasquet, L. Gutiérrez

**Affiliations:** 1 Centro Integral Oncológico Clara Campal (CIOCC), Madrid, Spain; 2 Instituto Catalán Oncología ICO-IDIBELL, Barcelona, Spain; 3 Hospital Universitario Puerta de Hierro, Madrid, Spain; 4 Hospital Virgen de La Salud, Toledo, Spain; 5 Clínica Universitaria de Navarra, Pamplona, Spain; 6 Corporació Sanitària Parc Taulí, Sabadell, Spain; 7 Biostatistics, Amgen Inc, Thousand Oaks, CA USA; 8 Health Economics, Amaris Consultancy, Barcelona, Spain; 9 Medical Department, Amgen S.A, Barcelona, Spain; 10 Pharmacoeconomics and Health Outcomes Research Department, Amgen S.A, Barcelona, Spain; 11 Unidad Gestion Clinica Oncologia Integral, Hospital Universitario Virgen del Rocio, Avda Manuel Siurot s/n, 41013 Sevilla, Spain

**Keywords:** Skeletal-related events, SRE, Bone metastases, Health care resource utilisation, Costs study

## Abstract

**Purpose:**

To estimate the cost per skeletal-related event (SRE) in patients with bone metastases secondary to solid tumours in the Spanish healthcare setting.

**Methods:**

Patients diagnosed with bone metastases secondary to breast, prostate or lung cancer were included in this multicentre, observational study. SREs are defined as pathologic fracture (vertebral and non-vertebral fracture), radiation to bone, spinal cord compression or surgery to bone. Health resource utilisation associated with these events (inpatient stays, outpatient, emergency room and home health visits, nursing home stays and procedures) were collected retrospectively for all SREs that occurred in the 97 days prior to enrolment and prospectively during follow-up. Unit costs were obtained from the 2010 eSalud healthcare costs database.

**Results:**

A total of 93 Spanish patients with solid tumours were included (31 had breast cancer, 21 prostate cancer and 41 lung cancer), contributing a total of 143 SREs to this cost analysis. Inpatient stays (between 9.0 and 29.9 days of mean length of stay per inpatient stay by SRE type) and outpatient visits (between 1.7 and 6.4 mean visits per SRE type) were the most frequently reported types of health resources utilised. The mean cost per SRE was between €2,377.79 (radiation to bone) and €7,902.62 (spinal cord compression).

**Conclusion:**

SREs are associated with a significant consumption of healthcare resources that generate a substantial economic burden for the Spanish healthcare system.

## Introduction

Patients with solid tumours are highly susceptible to developing bone metastases. The incidence of bone metastases is 65–75 % in patients with advanced breast and prostate cancer and 20–60 % in other solid tumours such as in the lung, bladder, kidney or thyroid [1].

In these patients, bone metastases are a common cause of morbidity or skeletal complications. These complications are referred to as skeletal-related events (SREs) and include: pathologic fractures (PF) [vertebral (VF) and non-vertebral (NVF)], radiation to bone (RB), spinal cord compression (SCC), and surgery to bone (SB) [2–5].

The incidence of SREs has been reported in several studies, mainly estimated through retrospective data collected in the placebo arms of the bisphosphonate clinical trials (previously considered to be the standard of care for the prevention of SREs) [6]. During the 2 years of follow-up, nearly 70 % of patients with breast cancer treated with placebo had ≥1 skeletal complication [1]. It is also known that patients who have previously suffered a SRE are at a higher risk of experiencing subsequent SREs [7–10]. SREs have a potential negative effect on the quality of life of patients [11–14], and can be associated with serious complications that can affect morbidity and mortality [15]. From a healthcare system perspective, suffering SRE is also related to increased health resource consumption that is directly related to increased medical costs [16].

In Spain there is an absence of studies that have analysed in-depth the health resource use associated with SREs. Being able to estimate the associated costs from this information is essential to perform rational allocation of resources across the Spanish healthcare system.

Between 2008 and 2010, a multicentre study (STARS) [17] was performed to establish health resource use associated with SREs in patients with bone metastases secondary to breast, prostate or lung cancer or bone lesions associated with multiple myeloma. A total of 478 patients with solid tumours from six countries (Canada, Germany, Italy, Spain, the United Kingdom and the United States of America) were included; 93 patients were recruited in Spain. Herein, we review the Spanish data set for the patients with solid tumours and describe the cost conversions used to estimate the cost by SRE type.

## Methods

### Primary objective and outcome measures

The primary study objective was to estimate the health resource utilisation associated with SREs by type of SRE and tumour type. The primary outcome measures included number and duration of patient stays; number of outpatient visits; number of emergency room and home health visits; and number and type of procedures.

### Study design

These analyses are based on the Spanish data collected in a multicenter, observational study (STARS). Patients were recruited between July 2008 and May 2010 and were followed for up to 18–21 months. Planned enrolment was 250 patients per country, with an annual attrition (drop out and death) assumed to be approximately 20 % for breast cancer and multiple myeloma (data not included in this analysis), 38 % for prostate cancer, and 55 % for lung cancer. Therefore, a country accruing 250 subjects had an expected total follow-up of 281 patient-year (including both patients with solid tumours and multiple myeloma).

As previously reported for the overall European cohort of this study by Bahl et al. [17], health resource utilisation data were collected prospectively for the duration of the subject’s participation in the study, and retrospectively through extraction of data from patients’ charts for all SREs occurring in the 97-day period before recruitment.

### Study population

Eligible patients were aged ≥18 years, diagnosed with bone metastases secondary to cancer of the breast, prostate or lung (per clinical practice at the participating centre), Eastern Cooperative Oncology Group (ECOG) performance status 0, 1 or 2 and at least one SRE within 97 days of providing signed informed consent. Patients were excluded if they had participated in a clinical trial for the treatment of bone metastases or had a life expectancy of less than 6 months (as determined by the treating physician). The study was authorised by the Independent Ethics Committee of each participating site and the Spanish Agency of Medicines and Medical Devices and was performed following the principles of the Declaration of Helsinki.

### Statistical analysis

All statistical analyses performed on the variables of resources used as a consequence of developing a SRE were descriptive. For continuous variables, the mean, median, standard deviation (SD) and minimum and maximum values were reported. For qualitative variables, the frequency and percentage were reported. These analyses were performed and summarised for all SRE types and outcome measures.

### Use of health resources

An electronic case report form was used to extract information about the health resources used, as listed above. Data were collected prospectively from patient enrolment and retrospectively (97 days) through reviewing medical charts and other relevant hospital and outpatient records. Attribution of health resource use associated with a SRE was performed by the investigators. In the event that patients experienced more than one SRE during the study period, the investigator determined which SRE any resource use should be allocated to. Radiation to bone or surgery to bone could be excluded from the health resource utilisation analyses if they were determined to be secondary to a primary SRE.

### Cost estimate by type of SRE

Patients with solid tumours were grouped in a single cohort. Costs of SREs were estimated from the standpoint of the National Health System; thus we only considered the direct costs derived from the management of the SREs, and the total cost was estimated per type of SRE. This cost includes the sum of all costs of health resources used by type of SRE collected. The unit costs of each resource (outpatient visits, hospital stay days, procedures, etc.) were obtained from the Spanish database of costs (eSalud) [19] (Table 1).

**Table 1:** Units costs used in cost conversion for Spain [18]

Type of resource		Single unit cost	
Hospital stay days by type of unit			
General unit		€252.84	
Intensive care unit		€1,094.50	
Oncology unit		€434.49	
Radiation therapy unit		€534.13	
Rehabilitation facility		€415.04	
Rehabilitation unit		€372.13	
Surgical unit		€403.51	
Others		€252.84	

Type of resource	Mean unit cost	Minimum cost	Maximum cost
Day hospital			
General unit (daily cost)	€182.43	€96.60	€290.30
Outpatient visits			
Emergency physician/nurse	€139.19	€49.36	€310.32
General surgery	€64.44	€48.34	€80.55
Nurse	€20.41	€10.24	€46.70
Oncologist	€79.35	€77.75	€80.94
Physical/occupational therapy	€11.06	€3.68	€20.13
Primary healthcare	€43.08	€19.51	€63.90
Radiation oncologist/radiotherapist	€102.10	€76.57	€127.63
Radiologist	€70.65	€52.98	€88.31
Emergency room visits			
Emergency room	€139.19	€49.36	€310.32
Home health visits			
Mean cost per visit	€64.25	€27.17	€127.80
Daily nursing home stay			
General unit (mean daily cost)	€133.14	€66.27	€161.73
Procedures completed during the outpatient visits			
Care of wounds/debridement	€1,538.39	€689.93	€2,234.09
Positron emission tomography/computerised tomography (PET-CT)	€1,148.29	€1,044.31	€1,233.14
Magnetic resonance imaging (MRI)	€241.91	€161.35	€611.18
Computed tomography (CT)	€169.02	€54.32	€323.87
Physical examination	€106.03	€93.79	€118.27
Radionuclides	€76.29	€53.57	€115.88
Laboratory tests	€56.93	€25.33	€117.23
X-rays	€31.66	€11.27	€67.94
External beam radiation therapy	€15.17		
Intensity-modulated radiation therapy (IMRT)	€15.17		

The costs were estimated at 2010 Euros, as the study reflects clinical practice between 2008 and 2010 and cost conversion analyses were conducted during 2011 (when cost data for 2010 were available).

### Cost conversion

The costs of SREs were estimated by associating the health resources attributed by the investigators to each type of SRE with the respective average unit costs (Table 1) through several formulae (one for each resource item). For example, the cost of hospital stays (Ch) for NVF was estimated using the equation: ${\text{Ch}}_{\text{NVF}} = \sum$ mean number facility stays_*u*_ × mean duration facility stays_*u*_ × daily cost_*u*_. Where *u* is the type of unit to which the patient with NVF was admitted (i.e. general unit, intensive care unit, etc.). These equations were validated by Oblikue Consulting (administrators of the eSalud cost database [19]) and by the authors of this publication.

The cost of PF was calculated as the weighted mean of the cost of VF and NVF, from the weight observed in the cohort of patients with breast, prostate and lung cancer.

## Results

### Baseline demographics

A total of 93 patients with solid tumours were recruited across 17 Spanish sites. Demographic characteristics are shown in Table 2. Patients experienced a total of 143 SREs (38 reported prospectively and 105 recorded retrospectively) in the health resource utilisation cost conversion analysis, distributed as follows: PF *N* = 25 (VF *N* = 10 and NVF *N* = 15), RB *N* = 96, SCC *N* = 15 and SB *N* = 7. Over 60 % of the patients in each cohort had experienced a SRE prior to retrospective collection period before enrolment although almost half of the patients recruited were receiving treatment with a bisphosphonate at or prior to recruitment. Mean patient follow-up was 7.1 months (SD 5.2) for patients with breast cancer, 5.9 months (SD 4.6) for patients with prostate cancer and 3.6 months (SD 3.8) for patients with lung cancer.

**Table 2:** Baseline characteristics and history of the disease by type of tumour

	Breast cancer (*N* = 31)	Prostate cancer (*N* = 21)	Lung cancer (*N* = 41)
Sex, *n* (%)			
Female	28 (90.3)	0 (0.0)	13 (31.7)
Male	3 (9.7)	21 (100.0)	28 (68.3)
Age (years)			
Median (range)	56 (38–79)	68 (50–86)	59 (35–80)
Age group, *n* (%)			
<65 years	20 (64.5)	7 (33.3)	26 (63.4)
≥65 years	11 (35.5)	14 (66.7)	15 (36.6)
ECOG performance status, *n* (%)			
0	6 (19.4)	6 (28.6)	7 (17.1)
1	12 (38.7)	10 (47.6)	17 (41.5)
2	13 (41.9)	5 (23.8)	17 (41.5)
History of SREs, *n* (%)^a^			
Yes	20 (64.5)	14 (66.7)	27 (65.9)
No	11 (35.5)	7 (33.3)	14 (34.1)
Time from the diagnosis of the primary tumour to recruitment (months)			
Median (range)	62.7 (0.6–259.9)	68.3 (0.6–261.2)	3.5 (0.3–49.1)
Time from the diagnosis of the bone metastasis to recruitment (months)			
Median (range)	4.86 (0.2–125.1)	11.10 (0.2–107.6)	1.81 (0.0–34.2)
Duration of prospective follow-up (months)			
Median (range)	6.87 (0.2–18.2)	4.63 (0.5–17.7)	2.60 (0.2–17.3)
Type of follow-up for all SREs^b^			
Prospective, *n* (%)	11 (23.4)	16 (44.4)	11 (18.3)
Retrospective, *n* (%)	36 (76.6)	20 (55.6)	49 (81.7)
Prior bisphosphonate use, *n* (%)	21 (67.8)	11 (52.4)	16 (39.0)
Time from first administration to recruitment (months)			
Median (Q1, Q3)	6.0 (2.5, 35.1)	5.5 (1.4, 8.6)	0.9 (0.4, 1.7)

*SREs* skeletal-related events, *ECOG* eastern cooperative oncology group

^a^Patients suffering any SRE in a period prior to recruitment of over 97 days

^b^Percentages calculated based on number of SREs

### Health resource utilisation

#### Hospitalisations

The percentage of SREs requiring hospital admission by SRE type were as follows: 100.0 % of SB, 73.3 % of SCC, 60.0 % of VF, 40.0 % of NVF, and 16.7 % of RB. Among those SREs requiring an inpatient stay, the longest mean duration of inpatient stay per inpatient stay was reported to be 29.9 days for VF, 21.6 days for SCC, 20.8 days for RB, 14.4 days for NVF and 9.0 days for SB.

#### Outpatient visits

RB was the SRE that required the highest percentage of outpatient visits (74.0 %), followed by VF (70.0 %), NVF (66.7 %), SCC (33.3 %) and SB (28.6 %). RB events also had the highest mean number of outpatient visits (6.4 visits per SRE). For the other SRE types, the mean number of visits was as follows: VF 2.8 visits; NVF 1.9 visits; SB 1.7 visits; and SCC 2.5 visits.

#### Visits to the emergency room

Visits to the emergency room were uncommon. The SRE requiring the most visits to the emergency room was SCC with an average of 0.3 visits per SRE. For the other SREs, excluding SB that did not require visits to the emergency room, the mean number of visits ranged from 0.0 to 0.2.

#### Procedures

Procedures included all tests and procedures completed during outpatient visits. The SRE requiring the highest number of procedures per SRE was RB with a mean of 6.4 procedures per SRE. In the outpatient setting, VF and SCC required 2.6 and 2.5 procedures per SRE, respectively, where as NVF and SB both required 1.7 procedures per SRE. External beam radiation therapy was the procedure most commonly used for the treatment of SREs, with a mean of 4.2, 1.1, 1.7 and 0.4 per RB, SCC, VF and NVF, respectively.

#### Home health visits and nursing home stays

With regard to home health visits and nursing home stays, only one case with SCC demanded this resource, with a stay of 103 days for the nursing home.

### Costs by type of SRE

Hospital stays were the main component of costs, comprising between 64 % of the total cost (in the case of RB) and 94 % (in the case of VF) (Table 3). The cost by SRE type for patients with solid tumours, estimated through cost conversion, ranged from €2,377.79 for RB, to €7,902.62 for SCC (Table 3).

**Table 3:** Breakdown of costs by type of SRE and resource used

	Non-vertebral fracture (%)	Vertebral fracture (%)	Radiation to bone (%)	Spinal cord compression (%)	Surgery to bone (%)
Hospital stay	€2,941.56 (92)	€6,560.57 (94)	€1,520.78 (64)	€6,609.24 (84)	€3,762.19 (88)
Outpatient visits	€135.12 (4)	€243.45 (3)	€639.29 (27)	€245.90 (3)	€58.30 (1)
Emergency room visits	€27.84 (1)	€27.84 (0)	€4.18 (0)	€45.93 (1)	€0.00 (0)
Home health visits	€0.00 (0)	€0.00 (0)	€0.00 (0)	€4.50 (0)	€0.00 (0)
Nursing home stay	€0.00 (0)	€0.00 (0)	€0.00 (0)	€914.23 (12)	€0.00 (0)
Procedures	€104.52 (3)	€136.33 (2)	€213.55 (9)	€82.82 (1)	€442.19 (10)
Total cost by SRE	€3,209.03	€6,968.18	€2,377.79	€7,902.62	€4,262.67

*SRE* skeletal-related event

VF represented 40 % of all PF and NVF the remaining 60 %. Table 4 shows the cost of the four SRE types described in this analysis, integrating the cost of VF and NVF into a single cost based on the above-mentioned weighting.

**Table 4:** Mean cost of SREs by type of SRE

Type of SRE	Mean cost
Pathologic fracture	€4,712.69
Radiation to bone	€2,377.79
Spinal cord compression	€7,902.62
Surgery to bone	€4,262.67

*SREs* skeletal-related events

## Discussion

This is the first study reporting a cost analysis of SREs in Spanish patients with bone metastases secondary to solid tumours based on data from a multicenter, observational study.

The data from this study illustrate that in addition to the well-reported devastating clinical burden that SREs impose on patients with metastatic bone disease, SREs are also associated with a substantial economic burden to the Spanish Healthcare system, as also reported in a retrospective database analysis conducted in Spain by Pockett et al. [16]. The vast majority of the associated health resource utilisation is derived from a requirement for inpatient stays (often of substantial duration) and outpatient visits as well as a substantial number of procedures. Of these resources, inpatient stays generally contribute the most to the cost of each SRE type. As might be anticipated due to the complicated nature of their treatment, SCC and VF were the SREs associated with the highest management costs (7,902.62€ and 6,968.18€, respectively), driven by the fact that the majority of them (73.3 and 60.0 %) required lengthy hospitalisations (with an average of 21.6 and 29.9 days per inpatient stay, respectively). Although hospitalisation was also required in all cases of SB, the average length of stay per inpatient stay was shorter (9.0 days) and thus the total cost of management was lower 4,262.67€ than that reported for SCC and VF. NVF had a cost of 3,209.03€ and RB was the SRE associated with a lowest management costs (2,377.79€), perhaps due to the fact that it is generally managed at ambulatory level (74.0 % of patients required 6.4 outpatient visits in average, and only 16.7 % required hospitalisation).

Our data are comparable to those reported by Pockett et al. [16], the only retrospective review of data published to date, which was based on the minimum basic data set of 28,162 cancer patients hospitalised during 2003 in Spain. This study also analysed the hospital burden associated with SREs in patients with breast, prostate or lung cancer and bone metastases. Mean hospital stay was reported to range from 12 to 20 days by SRE and tumour type, which is within the range observed in our study: from 9 days for SB to 30 days for VF per inpatient stay.

With regard to costs, Pockett et al. reported that for the first hospital admission due to a SRE, costs were €3,757, €3,585 and €4,298 (in Euros, of the year 2000), respectively, for patients with breast, prostate and lung cancer. These costs are in the range of those calculated in this study (between €2,377.79 for radiation to bone and €7,902.62 for spinal cord compression). However, it should be noted that in this study, costs should be higher than that reported by Pockett et al. mainly due to the fact that cost of outpatient visits and other costs are also included. Furthermore, our analysis has been conducted 10 years after that of Pocket et al. (year 2010 vs. 2000).

Our results may be conservative and underestimate the total burden of SREs. By study design, investigators directly attributed resources to the SREs. It is possible that not all investigators were able to access all records of resource use at all sites (for instance information about home health visits is not always shared between primary care physicians and hospitals). Furthermore, only health resources associated with SREs were investigated; pain requiring additional health resource use and lengthy inpatient stays was not considered as a SRE although evidence suggests that more than a third of the patients with bone metastases suffer severe pain [20] requiring hospital admission for analgesic titration of opioids or anaesthetic interventional techniques. Cost of treatment with bisphosphonates was also not included as their use was not specified in the study protocol and limited data on the dose and frequency of their administration were recorded. It should also be noted that direct non-healthcare costs or indirect costs such as transportation to/from hospital visits, payment of caregivers, sick leave, etc. were also not considered in this analysis. Similarly, patients with ECOG performance status >2 and overall survival <6 months were not included, despite the fact that they may arguably require more healthcare resources associated with their more advanced disease state.

Other important limitations of this study are associated with the difficulties to obtain information about some healthcare resources and associate them with a unit cost. An attempt was made to avoid double attribution of costs considering, for instance, only procedures performed in outpatient visits, as in the Spanish unit costs procedures performed in hospital admissions are already included in the price/day of stay.

One aim of this cost conversion was to calculate the mean cost of SREs by type of tumour. A total of 31 patients with breast cancer, 21 with prostate cancer, and 41 with lung cancer were included in the study, experiencing a total of 143 SREs included in the cost analysis. Due to the low number of events when patients were separated by tumour and SRE type, it was decided to calculate aggregated resource use and costs by type of SRE for all solid tumours, assuming that the resource use and costs, for instance, for SB were the same in patients with breast, prostate or lung cancer. This assumption was later confirmed by the results of this study. Despite this, one potential weakness of this analysis was the limited number of SREs included for some SRE types, mainly SCC and SB. Nevertheless, these results concluded that hospitalisation is the main cost driver across all SREs, which was confirmed by the results of the overall European analysis of the STARS study, which included a total of 893 SREs [17].

Despite the possible limitations associated with observational studies, data from other research support the underlying fact that all SREs are a major burden for patients with regard to worsening health quality, need for hospital admissions, impairment of physical and emotional health and reduced survival [14, 21, 22]. Notably, patients experienced multiple skeletal complications even during the short follow-up of the study (38 prospective SREs reported for 93 patients with mean follow-up of approximately 4–7 months, varying by tumour type), which further illustrates the substantial burden of disease. Thus, preventing SREs using the most appropriate interventions is important to achieve a considerable reduction in patient burden as well as potentially reducing the requirement for costly hospitalisations and decreasing associated treatment costs across the Spanish national health system.
